# Household air pollution and blood pressure among adult women participants of the Household Air Pollution Intervention Network Trial: An exposure-response analysis

**DOI:** 10.1016/j.envres.2025.122570

**Published:** 2025-08-12

**Authors:** Ajay Pillarisetti, Wenlu Ye, Jennifer L. Peel, Howard Chang, Lindsay J. Underhill, Kalpana Balakrishnan, Anaité Díaz-Artiga, John P. McCracken, Ghislaine Rosa, Lisa M. Thompson, Vigneswari Aravindalochanan, Dana Boyd Barr, Yunyun Chen, Marilú Chiang, Maggie L. Clark, Victor Davila-Roman, Shirin Jabbarzadeh, Michael A. Johnson, Miles A. Kirby, Amy E. Lovvorn, Luke P. Naeher, Florien Ndagijimana, Ricardo Piedrahita, Naveen Puttaswamy, Lance A. Waller, Jiantong Wang, Kendra N. Williams, Laura Nicolaou, William Checkley, Thomas F. Clasen, Joshua P. Rosenthal, Kyle Steenland, HAPIN Investigators

**Affiliations:** a University of California, Berkeley, CA, USA; b World Health Organization, Geneva, Switzerland; c Colorado State University, Fort Collins, CO, USA; d Emory University, Atlanta, GA, USA; e Washington University in St. Louis, St. Louis, MO, USA; f Sri Ramachandra Institute for Higher Education and Research, Chennai, India; g Universidad del Valle de Guatemala, Guatemala City, Guatemala; h University of Georgia, Athens, GA, USA; i University of Liverpool, Liverpool, UK; j PRISMA, Lima, Peru; k Berkeley Air Monitoring Group, Berkeley, CA, USA; l Harvard T.H. Chan School of Public Health, Boston, MA, USA; m Eagle Research Center, Kigali, Rwanda; n Johns Hopkins University, Baltimore, MD, USA; o Fogarty International Center, NIH, Bethesda, MD, USA

**Keywords:** Particulate matter, Black carbon, Carbon monoxide, Household energy, Solid fuel, Intervention study

## Abstract

**Background:**

Exposure to air pollutants, like fine particulate matter (PM_2.5_), has been linked to higher blood pressure (BP). Few studies have examined this association in biomass-dependent settings. We seek to determine whether high exposure during a 16 month period was associated with an increase in BP among older adult women over the study period and to determine whether short-term increases in exposure were associated with higher coincident blood pressure.

**Methods:**

As part of a randomized controlled trial of a free liquefied petroleum gas cookstove and 18-month fuel supply, we measured BP and personal exposure to PM_2.5_, black carbon (BC), and carbon monoxide (CO) on 368 adult women (mean age 52) in four countries (Rwanda, Peru, Guatemala, and India). We considered short- and long-term associations, the latter measured by change in BP and the former in mixed models of repeated measures.

**Findings:**

We found an association between short-term exposure and both systolic and diastolic BP. The highest quartile of BC was associated with a 2 mmHg increase compared to the referent group (p = 0.03). We also found a positive association with PM_2.5_, where the highest quartile had a 1.6 mmHg increase in SBP versus the referent group (p = 0.05). We found no association with long-term exposure, nor between CO and BP.

**Interpretation:**

In settings where HAP dominates air pollution exposure, we found short-term exposure to BC and PM_2.5_ associated with increased BP, consistent with past literature. The lack of long-term associations may have been due to insufficient follow-up.

**Funding:**

The study is registered with ClinicalTrials.gov (NCT02944682) and funded by the U.S. National Institutes of Health (1UM1HL134590) in collaboration with the Bill & Melinda Gates Foundation (OPP1131279).

## 1. Introduction

High blood pressure is the leading cause of mortality and morbidity in men and women globally, including in lower- and middle-income countries (LMICs)(Brauer et al., 2024). BP is highly variable and can be affected by diet (Forman, 2009), physical activity (Brook, 2017), and various environmental factors (Arroll and Beaglehole, 1992), including temperature, noise, and exposure to air pollution (Rajagopalan et al., 2024).

Solid biomass (e.g., wood, charcoal, crop residues, animal dung, and coal) is used as a cooking fuel (IEA et al., 2023) regularly by between 2 and 3 billion people worldwide. Burning these fuels in inefficient cookstoves, often in poorly ventilated kitchens, results in high exposure to household air pollutants, including PM_2.5_ (particulate matter with a diameter of <2.5 μm), CO (carbon monoxide), and BC (black carbon). The 2021 Global Burden of Disease attributed approximately 3.2 million deaths to household air pollution exposure (Brauer et al., 2024). Short-term (i.e., hours to days) exposure to ambient PM_2.5_ has been linked to increased BP (Brook et al., 2004, 2010); chronic exposure can promote development of hypertension (Brook et al., 2011). Biologically, it is hypothesized that exposure to PM can alter the autonomic nervous system and lead to systemic inflammation and upregulation of vasoconstrictors (Brook and Rajagopalan, 2009; Feng et al., 2023).

Studies of HAP exposure and BP have been inconsistent but are indicative of elevated blood pressure with increasing exposure. Studies from China (Baumgartner et al., 2011, 2014, 2018; Chen et al., 2020; Lee et al., 2012; Lin et al., 2021; Yan et al., 2016; Yu and Zuo, 2022; Zheng et al., 2021), Bangladesh (Khan et al., 2021), Peru (Burroughs et al., 2015; Kephart et al., 2020; Painschab et al., 2013), Honduras (Young et al., 2018), India (Aung et al., 2018; Dutta et al., 2011; Dutta and Ray, 2014), Nigeria (Ofori et al., 2018), Bolivia (Alexander et al., 2015), and Guatemala (McCracken et al., 2007) all suggest that biomass fuel use elevates BP and/or increases hypertension risk. Other studies, contrastingly, have found insignificant associations or *lower* BP among solid fuels users in multicountry evaluations (Arku et al., 2018) and in single country studies in Nepal (Neupane et al., 2015; Tiwana et al., 2020), China (Clark et al., 2019), Pakistan (Fatmi et al., 2014; Jamali et al., 2017), India (Chakraborty and Mondal, 2018; Norris et al., 2016), and Nicaragua (Clark et al., 2013). This inconsistency might be due to exposure misclassification; potentially biased, self-reported outcomes (e.g., hypertension); and/or weaker study designs (e.g., cross-sectional evaluations).

The multi-country Household Air Pollution Intervention Network (HAPIN) randomized controlled trial assessed the health effects of an LPG stove and continuous fuel supply intervention to replace primary biomass stoves. The trial was shown to have fidelity and adherence to the intervention (Quinn et al., 2021; Williams et al., 2023) and led to substantial reductions in exposure (Johnson et al., 2022; Ye et al., 2024; Pillarisetti et al., 2023). The HAPIN trial enrolled pregnant women to study birthweight, infant growth, and infant pneumonia. In a subset, an older woman living with the pregnant participant was also enrolled.

One of the primary endpoints of HAPIN was the effect of HAP on blood pressure among these older women (mean age 52). We hypothesized that both acute and longer-term exposure to these pollutants would be associated with higher BP. Prior HAPIN cross-sectional analyses of baseline BP and pollutant levels among older women did not find an overall association of pollutants and BP, but did find a positive association between PM_2.5_ and BP among women older than age sixty (Nicolaou et al., 2022). Here, we present exposure-response results from analyses of both short- and longer-term exposure to PM_2.5_, BC, and CO and their association with BP across approximately 16 months of follow up for 368 older women.

## 2. Methods

### 2.1. Study design and population

The HAPIN trial was conducted in four LMIC settings (Tamil Nadu, India; Jalapa, Guatemala; Puno, Peru; and Kayonza, Rwanda). The study design and population have been described previously (Barr et al., 2020; Clasen et al., 2020; Johnson et al., 2020). We enrolled ~3200 biomass-using households (~800 in each country) with a pregnant woman (aged 18 to <35 years, 9 to < 20 weeks of gestation). In 418 of the households, we also enrolled a second, non-pregnant adult woman (aged 40 to <80 years) to assess cardiopulmonary and other outcomes. Participants were excluded if they were active smokers, pregnant (via self-report), or planned to move out of their current households in the next 12 months.

We randomly assigned half of the households (with and without a 2nd non-pregnant adult woman) to an intervention group that received LPG cookstoves and an 18-month supply of LPG. The control group continued cooking with biomass and chose to either receive the same intervention package upon the completion of the study or to receive other compensation for participation (Quinn et al., 2019). Images of common biomass and LPG stoves in each country are provided in Supplemental Fig. S6.

Among this population, we conducted repeated measures of 24-h air pollution exposure (PM_2.5_, BC, and CO) and BP, timed to match measurements performed among pregnant participants in the same household: once at baseline (pre-intervention, 9 to < 20 weeks of gestation), twice during the pregnancy (24–28 weeks and 32–36 weeks of gestation), and three times during the child’s first year of life (<3 months, ~6 months, and ~12 months). The non-pregnant older adult women are the study population of the current analysis.

### 2.2. Personal exposure monitoring

As described elsewhere, we measured PM_2.5_, BC, and CO 24-h personal exposures up to six times per participant. During each exposure monitoring session, participants wore customized garments with the instrumentation attached and close to their breathing zone. Personal exposure to PM_2.5_ was measured using the Enhanced Children’s MicroPEM (ECM, RTI International, Research Triangle Park, NC, USA). The ECM measures PM_2.5_ continuously with a nephelometer and collects a gravimetric sample by drawing air through an impactor attached to a cassette containing a 15 mm Teflon^®^ filter (PT15-AN-PF02; Measurement Technologies Laboratories, Minneapolis, MN, USA) (Johnson et al., 2020). Gravimetric concentrations were used in the analysis when available; when not, adjusted instrument-specific nephelometric concentrations were used. We characterized BC levels on PM_2.5_ filters using transmissometry with the SootScan^™^ Model OT21 (Magee Scientific, Berkeley, CA, USA). BC depositions were estimated using previously published methods (Garland et al., 2017); tranmissometry was performed at the University of Georgia (UGA, Athens, GA, USA) for samples collected in Guatemala, Peru, and Rwanda and at Sri Ramachandra Institute for Higher Education and Research (SRIHER, Chennai, India) for samples collected in India. Personal CO exposure was measured using the Lascar EL-USB-300 (Lascar Electronics, Erie, PA, USA), a small CO datalogger (detectable range of 0–300 ppm) that logs concentrations continuously. Participants wore a vest or apron containing the sampling equipment during the 24-h measurement period, except when sleeping, bathing, or when conducting other activities which could damage the equipment or impede participant movement. During these times, the vest or apron was kept nearby. Sociodemographic, household characteristics, and activity pattern data were collected. Detailed exposure monitoring procedures and data quality control and assurance (Johnson et al., 2020) and the exposure assessment findings have been described previously (Johnson et al., 2022; Ye et al., 2024; Pillarisetti et al., 2023). Briefly, gravimetric data were validated using the following approach: technicians (A) evaluated flow rates before and after sampling with a flowmeter enabling removal of samples beyond expected ranges; (2) marked damaged filters as invalid; and (3) removed data considered invalid that did not meet QA criteria, including sampling duration (24 h ± 6 h), flow rate (300 ± 100 mL/min), and inlet pressure (95th percentile, <5 in. H2O). 690 field blanks were collected and country-specific median blank correction was performed. Filters removed from gravimetric analysis were not analyzed for BC; an additional outlier removal step for values outside of threshold ranges (0–100 μg BC) was applied.

CO monitors were calibrated using zero air and 40–80 ppm CO span gas. Traces from CO loggers were checked automatically at regular intervals via an online QA procedure and were manually visually inspected and rated. Data beyond duration bounds (24 h ± 6 h) or flagged during manual visual inspection were considered invalid and removed.

### 2.3. Blood pressure measurements

Resting BP was measured on the right arm by a nurse or trained field worker in the morning following 24-h personal exposure monitoring. BP was measured in triplicate (with at least 2 min between measurements) using an automatic monitor (Omron^®^ Model HEM-907XL); the average of the readings was used for data analysis (Clasen et al., 2020; Ye et al., 2022). The participant was instructed to sit on a chair in a quiet room for 5 min with legs uncrossed, their back supported by the chair, and their arm supported on a table before starting the measurement. Fasting was not required. The participant confirmed that she had not smoked, consumed alcohol/caffeinated beverages, or cooked using biomass in the past 30 min. If she had done any of those activities, she was asked to refrain from doing these activities for 30 min before proceeding with the measurements. A participant with a measured 1) SBP ≥140 mmHg and/or DBP ≥90 mmHg, or 2) SBP <80 mmHg or DBP <40 mmHg was referred to the nearest health facility to receive age-appropriate treatment. SBP values < 70 mmHg and DBP values < 35 mmHg were excluded as implausible. We also calculated pulse pressure (PP=SBP−DBP) and mean arterial pressure (MAP=DBP+(SBP−DBP)∕3).

### 2.4. Statistical analysis

Statistical analyses were decided upon before the analysis and published with the trial registration (HAPIN Investigators). We modeled exposure-response relationships between exposures to PM_2.5_, BC, and CO (separately) and the primary (systolic BP) and secondary (diastolic BP) outcomes.

We used separate models to estimate the short- and long-term associations between exposure and blood pressure. First, we estimated a “change-score” model which evaluated associations over the 16 month follow-up period. Second, we used a repeated measures model to evaluate whether a) the long term average PM2.5 exposure affects BP across repeated measures and b) whether short term exposure increases BP.

We considered models with linear, log-linear, and quartiles exposure terms. We evaluated prediction errors and identified the most parsimonious model based on goodness-of-fit metrics (i.e., R^2^, plotting observed and predicted values, use of residual plots, and added variable plots) and Akaike Information Criteria (AIC).

We first estimated the association between long-term exposure and BP outcomes using the regression model given by Eq. 1

Eq. 1 $$E[d_{i}]=\beta_{0}+\beta_{1}X_{i}+\gamma Z_{i}$$

where $d_{i}$ is the change in outcome (SBP/DBP) between the 6th follow-up measurement (~18 months post-intervention) and the baseline measurement (baseline-final BP); $X_{i}$ is the time-weighted average exposure of interest (PM_2.5_/BC/CO) across the follow-up period, and $Z_{i}$ is the vector of confounders. The time-weighted average exposures were estimated using 24-h personal measurements at baseline and all follow-up visits (up to five per participant). For participants in the control group, an average was calculated from all available measurements. For participants in the intervention group, baseline exposure levels were weighted by days prior to LPG installation, while the average of postbaseline measurements was weighted by the days after LPG installation. The time-weighted average gives more weight to the baseline measurement for those in the intervention group for whom the intervention was received later. Participants were excluded from the analysis if the baseline measurement was missing. This model evaluates the change in BP from baseline to end of follow-up, assessing the cumulative effect of exposure over the whole period. Using the change-score accounts for baseline BP without putting it in the model, thus avoiding any impact of exposure on baseline BP (Glymour et al., 2005).

In a second approach, we used a mixed-effects model with a random intercept for each individual to assess the short-term association between repeated BP measurements and PM_2.5_, BC, or CO exposures (Eq. (2)).

Eq. 2 $$E[y_{ij}]=\beta_{0}+\theta_{i}+\beta_{1}X_{i}+\beta_{2}(X_{ij}-X_{i})+\gamma Z_{i}$$

where $y_{ij}$ denotes the outcome for participant i at visit j, $\theta_{i}$ is the participant-specific random intercept. $X_{i}$ is the time-weighted average of all available measurements as described above, and ($X_{ij}-X_{i}$) is the participant-specific deviation from average exposure $X_{i}$ for exposure at time point j. The parameter $\beta_{1}$ reflects the ‘long-term’ or average effect of exposure across the study period, while the parameter $\beta_{2}$ describes the within-participant short-term association between exposure and outcome, adjusted for impacts of long-term exposures (both have been found in studies of ambient PM_2.5_).

This model contains two special cases: 1) when $\beta_{1}=\beta_{2}$, the model reduces to a random-intercept model that only uses the time-varying exposure directly and 2) when $\beta_{2}=0$, the model reduces to a random-intercept model with only time-weighted average exposure. In sub-analyses, we considered models with only time-varying exposure – that is, without average exposure in the model, thus evaluating only the short-term impact of exposure on blood pressure.

Confounders and covariates included in the model are listed in Supplemental Table S1 and were chosen a priori based on a review of the literature. Study site (IRC) was known to be associated with both BP and exposure. Age, BMI, time of day of measurement, and markers of socioeconomic status (education, diet diversity, food insecurity index) have been found in the literature to be strongly associated with blood pressure, were potentially associated with exposure, and were included in all models. We assessed effect modification by age (continuous and dichotomous by the median), IRC (categorical: Guatemala, India, Peru, and Rwanda), BMI (continuous and categorical: underweight, healthy weight, and overweight/obese), and study arm (categorical: control vs. intervention) on the additive scale in linear models by including interaction terms between exposure and these variables. As a sensitivity analysis, we ran models with the entire dataset (not excluding participants on medication).

All primary, secondary, and sensitivity analyses were conducted using SAS (SAS, 2020) and R (version 4.2.2).

### 2.5. Ethics review and trial registration

The study protocol was reviewed and approved by institutional review boards (IRBs) or Ethics Committees at Emory University, Johns Hopkins University, Sri Ramachandra Institute of Higher Education and Research and the Indian Council of Medical Research – Health Ministry Screening Committee, Universidad del Valle de Guatemala and Guatemalan Ministry of Health National Ethics Committee, Asociación Benefica PRISMA, the London School of Hygiene and Tropical Medicine and the Rwandan National Ethics Committee, and Washington University in St. Louis. The study has been registered with ClinicalTrials.gov (Identifier NCT02944682).

## 3. Results

### 3.1. Household and participant characteristics at baseline

After removing participants that were a) pregnant (n = 8), b) taking antihypertensive medication during any time of the study (n = 41), and c) determined ineligible post-randomization (n = 1), 368 women and a total of 1626 observations were left in the exposure-response analysis.

Baseline participant and household characteristics study-wide and by study site are summarized in Table 1. The participants were 40–74 years of age (mean age, 51.6 years). The mean (SD) BMI was 25.4 (5.2) kg/m^2^; 34 % were overweight (BMI, 25–29.9 kg/m^2^), and 17.9 % were obese (BMI,≥30 kg/m^2^). The relatively high proportion of overweight and obese was driven by Guatemala and Peru, where 55.3 % and 81.6 % of the participants fell into these respective categories. Most participants had no formal or incomplete primary education (79.3 %). Most of the women in India (75.0 %) and Rwanda (78.4 %) worked in agriculture, while most women were not employed outside of the home in Guatemala (96.5 %) and Peru (73.6 %). Although the participants were non-smokers, 12.5 % (mainly in Guatemala and India) were exposed to secondhand tobacco smoke in their households. The main cooking fuel used in Guatemala, India, and Rwanda was wood (94.6 %–100 %), while the reported primary household fuel in Peru was cow dung (88.0 %).

### 3.2. Personal exposure to PM_2.5_, BC, and CO

We obtained 1395 valid 24-h personal PM_2.5_ measurements, 1197 valid BC measurements, and 1236 valid CO measurements from the 368 participants included in this analysis. A full description of exposure findings for this population has been published previously (Ye et al., 2024). Mean exposures were 77.1 μg/m^3^ (SD 72.2) for PM_2.5_, 8.1 μg/m^3^ (SD 6.3) for BC, and 1.7 ppm (SD 2.0) for CO. The intervention reduced exposures substantially: 59 % for PM_2.5_, 60 % for BC, and 73 % for CO. Median exposures to PM_2.5_ were 73.5 μg/m^3^ in the control group and 29.4 μg/m^3^ in the intervention group. Similar reductions were noted for CO (1.42 ppm control, 0.48 ppm intervention) and BC (9.4 μg/m^3^ control, 2.82 μg/m^3^ intervention). PM_2.5_ and BC exposures were strongly correlated (Spearman’s ρ = 0.77); PM_2.5_ and CO were relatively weakly correlated (ρ = 0.45), as were CO and BC (ρ = 0.37). Reductions of all pollutants were stable over time. Detailed findings from the exposure assessment are under revision and include additional data on sociodemographic characteristics, plots of exposure over time, and missingness. Overall, 11 % of PM_2.5_, 20 % of BC, and 19 % of CO were either missing or invalid, and were excluded from the analysis.

Average BP throughout the study was 113.2 (SD 14.1) systolic and 69.0 (SD 9.9) diastolic. Supplemental Fig. S1 contrasts the distribution of SBP and DBP over time. There was no clear pattern in overall SBP or DBP during the follow-up visits, although both SBP and DBP showed an increase toward the end of the trial. Similar trends were observed across IRCs (Fig. S2-S5).

About 35% of participants had BP levels classified as hypertension stage 1 or 2 (henceforth “hypertensive”) at least one point throughout the study. At baseline, 21.2% (n = 77) of participants were hypertensive (SBP >130 mmHg or DBP >80 mmHg). Post-intervention, approximately 15% of participants were hypertensive. Supplemental Table S3 summarizes the number and proportion of participants in each American Heart Association (AHA) BP category and study period.

We further examined BP trends in participants <50 and ≥50 years, given biological changes and relatively increased cardiovascular risk beginning in the fifth decade of life (Baumgartner et al., 2011; Mackay and Mensah, 2004). Though statistically insignificant, mean SBP was slightly lower among women ≥50 years in the intervention group, except for the last visit. We did not find similar trends among women <50 years or in DBP.

### 3.3. Exposure-response analysis

Table 2 reports the results of the change-score analysis for untransformed and log-transformed continuous exposures. We found no change-score coefficient to be statistically significant at the 0.05 level. The quartile analyses of change-scores (Supplemental Table S4) were consistent and showed no association.

**Fig. 1.**
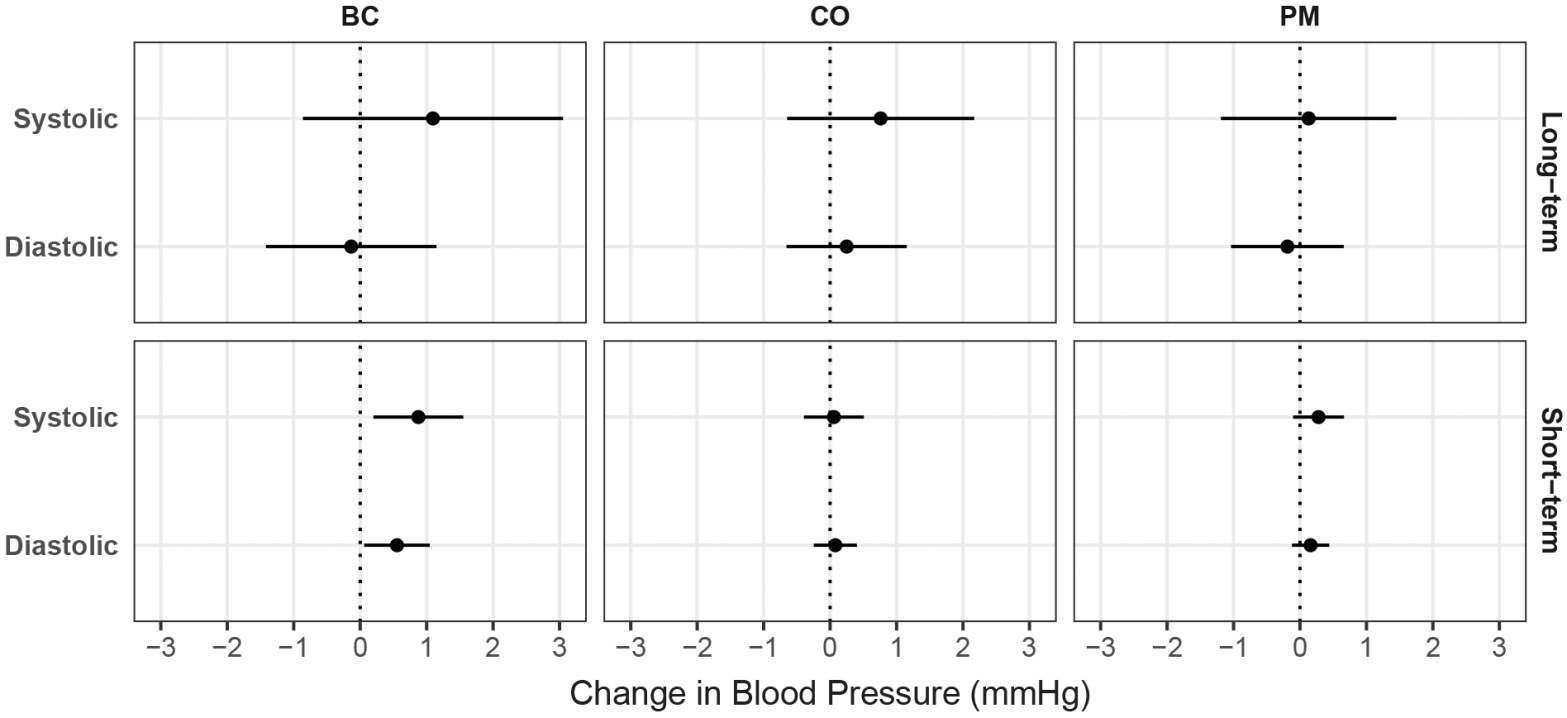
Changes in systolic and diastolic blood pressure associated with an IQR increase in pollutant exposures. BC = Black Carbon (μg/m^3^); CO = Carbon Monoxide (ppm); PM = PM_2.5_, particulate matter with an aerodynamic diameter of ≤2.5 μm (μg/m^3^). Error bars are 95 % confidence intervals. Mixed models adjusted for age, education, diet diversity, BMI, food insecurity, time of day, and IRC. The long-term panels estimate exposure as the time-weighted mean pollutant level over the entire follow-up. The short-term values estimate the average change from the mean. IQRs for PM_2.5_, BC, and CO are 78.9 μg/m^3^, 10.1 μg/m^3^, and 2.1 ppm, respectively.

Table 3 and Fig. 1 report findings from repeated measures analysis using mixed models. Models include average exposure during follow-up in the model and short-term change at each visit from the long-term average. In this analysis, no association between long-term average exposure was found to be statistically significant at the 0.05 level, neither when untransformed nor log-transformed. However, for BC, we found an association between short-term increased SBP and DBP with higher BC exposures measured at the time of visit, i.e., at the same time the BP was measured.

To investigate this further, we removed the long-term average from the model and ran the same model for short-term changes for untransformed, log-transformed, and quartile analyses. Here, we again found statistically significant short-term effects of BC for both SBP and DBP, with increasing BC exposures associated with increased BP. The highest quartile of BC was associated with an increase of about 2 mmHg to SBP. The highest quartile of PM_2.5_ was also found to have an elevation of 1.6 mmHg (p = 0.05). Short-term BC and PM_2.5_ were highly correlated (Spearman’s ρ = 0.82). The intra-class correlation in these models was between 0.50 and 0.60, indicating greater variation between women than within women. No marked effects were seen for CO (not shown), as expected from Table 3 and Fig. 1. The squared correlation coefficient (R-square) for the linear model for SBP was 0.75 for the PM_2.5_ model and 0.76 for the BC model.

In secondary analyses, we considered effect modification by age, study site, and BMI (Supplemental Table S5). In change score models, finding were inconsistent across study sites, although Guatemala has a significant increase in BP over time with higher PM_2.5_ (p = 0.002) and higher BC (p = 0.002). Long-term average effects were not significant in any sub-group. In short-term models, consistent with our primary findings (and earlier baseline findings (Quinn et al., 2021)), we found a significant association between BC on SBP among participants older than the median age of 50 (p = 0.03). Short-term associations were also stronger among those with lower BMI (p = 0.01). However, neither of these interactions (age, BMI) were significant at the 0.05 level when interactions terms were added to the models. Positive associations for short-term exposures were consistent across all IRCs. As sensitivity analyses, we ran both change-score and mixed models (with long-term average and short-term change) for SBP for both PM_2.5_ and BC after adding back the 41 women who had been excluded for taking BP medication. There were no marked changes in results, and again only the short-term change with BC was significant at the 0.05 level (results not shown). We also added secondary smoke (passive smoking) to models for both long-term and short-terms effects, but found it was neither an important predictor of blood pressure nor did it change the association with pollutant exposure.

In other secondary analyses, we also ran linear models for all three pollutants with mean arterial pressure (MAP) and pulse pressure (PP) as outcomes (Supplemental Tables S6 and S7). We included change score models, models with repeated measures with short term exposure in the model, and models with and without long-term exposure. Patterns were similar to results for SBP and DBP in Table 4; significant (at the 0.05 level) short term increases in both MAP and PP were found in relation to BC exposure. Finally, to account for potential impacts of seasonality, we included a term for cold months as a proxy for winter in our models. We re-ran models including a variable for winter (defined separately for each country). Cold months predicted higher BP in our adjusted model, but t-tests found no significant differences in either PM_2.5_ or BC levels during winter months. Including the winter term had little effect on the associations with exposure (Supplemental Table S8).

## 4. Discussion

Our short-term findings are consistent with previous HAP literature (see Supplemental Table S9). For the relationship between personal PM_2.5_ exposure and BP, studies in China have shown a consistent positive relationship with short-term higher exposure associated with higher BP. Baumgartner et al. found that a 1-ln-μg/m^3^ increase in PM_2.5_ exposure was associated with a short-term 2.4 mmHg higher SBP among Chinese adult women living in rural Sichuan province; in separate work, they reported that a 1-ln-μg/m^3^ increase in PM_2.5_ was associated with 2.2 (95 % CI: 0.8, 3.6) mmHg higher SBP and 0.5 (95 % CI: −0.3, 1.3) mmHg higher DBP among Chinese adult women in Yunnan province^12 14^. The more recent INTERMAP China Prospective found a short-term 1-ln-μg/m3 increase in PM_2.5_ exposure was associated with 1.5 (95 % CI: 0.2, 2.7) mmHg SBP and 1.0 (95 % CI: 0.4, 1.7) mmHg DBP.

The PM_2.5_-BP relationship reported in other countries is less consistent. In the large, multi-country PURE study, PM_2.5_ exposure was positively but non-significantly associated with SBP when comparing Q4 with Q1 of exposure (2.15 mmHg, 95 % CI: −0.59, 4.9) and with DBP (1.35 mmHg, 95 % CI: −0.2, 2.89) (Arku et al., 2020). A cross-sectional study in Honduras did not find a significant association between short-term PM_2.5_ exposure and BP: a 1-log-μg/m^3^ increase in personal PM_2.5_ exposure was associated with 0.8 (95 % CI: −2.2, 3.8) mmHg SBP and 0.4 (95 % CI: −2.0 to 2.7) mmHg DBP (Young et al., 2018). The CHAP trial in Peru also found no consistent exposure-response relationships between PM/BC/CO and BP (Checkley et al., 2021), while the GRAPHS trial in Ghana found a positive association between exposure to CO and DBP: for a 1 ppm increase in CO exposure, DBP was 0.43 mmHg higher (0.01, 0.86) (Quinn et al., 2016).

The lack of impact on long-term blood pressure may be the result of insufficient follow-up, exposure measurement error, or the relatively healthy nature of the cohort (as assessed by the high fraction of participants that were normotensive throughout the trial). We also note that this was a relatively young population to see BP changes over time.

Systematic reviews and meta-analyses have synthesized evidence of the relationship between long- and short-term exposure to ambient air pollutants (including PM2.5) and hypertension (Cai et al., 2016), as well as both hypertension and blood pressure (Yang et al., 2018). In the more recent review by Yang et al., short-term exposure was defined as < 30 days, while long-term is ≥ 30 days. That review found that no association between long-term PM2.5 exposure and SBP, and a significant association with DBP, but noted ‘extreme heterogeneity’ in findings. For short-term exposures, their meta-analysis found significant associations between PM2.5 and both SBP and DBP, again with high heterogeneity. They also found in sensitivity analyses that the aforementioned relationships were not robust. Based on GRADE quality of evidence ratings, associations were considered of low or very low confidence.

In that review, exposure assessment strategy is reported; only a very small fraction of air pollution exposures were assessed using personal monitors. For most studies reviewed, either the nearest monitoring station or a modeled exposure were used. This different from our approach, where we have repeated measures of personal exposure, which have been shown to be relatively stable over time (Steenland et al., 2025). Definitions of short- and long-term exposure were inconsistent between the two most recent systematic review and meta analyses; in the earlier review by Cai et al., short-term was defined as “over several days” while long-term assessed “average exposure … over years.” We note that for many of these studies, regardless of exposure duration, exposures are much lower than what was noted in our contexts, where household sources contribute substantially to overall exposure.

The inconsistency of our findings mirrors the literature broadly, indicating that further research may be needed to disentangle which exposures, and at what levels, may impact blood pressure. This could include investigation of PM components, like metals, exposure to which may be associated with changes in inflammatory states (Gu et al., 2024) and thus impact blood pressure, and further work understanding if there are threshold effects, both in terms of exposure duration and intensity, for the impact of air pollution on blood pressure. We note that some air pollutants, like NO, are vasodilators; thus the total impact of a mixture of air pollutants, like household air pollution, may not be straightforward or explained simply by pollutants that serve as a proxy for the mixture.

Finally, unlike the studies in Ghana, we did not find an association between CO and blood pressure. That study focused on pregnant women, unlike our analysis. Exposure levels were comparable between the current study and the trial in Ghana; further research is needed to better understand how and at what level CO might impact blood pressure, and if that impact is influenced by life course events, like pregnancy.

There are several limitations to our study. While we measured exposure repeatedly, some amount of measurement error is possible, though likely non-differential and biasing toward a null relationship. On the other hand, analysis of a subset of mothers and children with double the amount of samples found no difference between regular (per protocol) and supplemental samples, suggesting our number of samples was sufficient to capture the long term average (Steenland et al., 2025). We note that many relevant variables of interest – including salt consumption, other dietary parameters, local ambient temperature, and family history of cardiovascular disease – were not captured and may play an important and unmeasured role and modulate the impact of exposure on BP (Liu et al., 2025).

Our short-term findings indicate some potential benefits of HAP-reducing interventions and policies. Future work would benefit from longer follow-up and restriction to older women, and more full consideration of the multiplicity of risk factors that influence BP and cardiovascular health.

## 5. Conclusions

In this exposure-response analysis involving non-pregnant adult women from four diverse LMICs, we found a positive association between short-term exposure and BP at a given visit prior to subsequent BP measurement for both BC and PM_2.5_. Uppermost quartiles were statistically significant for SBP when compared to the lowest quartile, with increases of 2.0 and 1.7 mmHg SBP, respectively. We found that no relationship between exposure to measured air pollutants and longer-term SBP or DBP. While such increases in BP with higher shorter-term exposure BC and PM2.5 are not clinically relevant at an individual level, they might be important at a population level.

## Supplementary Material

Pillarisetti_EnvRes_2025_SI

## Figures and Tables

**Table 1:** Characteristics of the participants and households at baseline^a^.

	Guatemala	India	Peru	Rwanda	Overall
	n = 114	n = 92	n = 125	n = 37	n = 368
*Participant Characteristics*					
Age					
Mean (SD)	52.6 (7.7)	49.1 (6.5)	52.4 (7.6)	51.9 (8.3)	51.6 (7.6)
Range	40.4–73.8	40.2–71.6	40.1–73.6	40.5–70.7	40.1–73.8
Systolic Blood Pressure (mmHg, mean (SD))	116.1 (16.7)	121.8 (14.1)	107.3 (11.5)	116.8 (12.5)	114.6 (15.1)
Diastolic Blood Pressure (mmHg, mean (SD))	69.2 (10)	76.9 (9.6)	64 (8.7)	72.2 (8.7)	69.7 (10.6)
Body Mass Index (BMI) (kg/m^2^), n (%)^b^					
Mean (SD)	25.8 (4.3)	20.9 (3.4)	28.9 (4.4)	23.1 (4.2)	25.4 (5.2)
Underweight (<18.5)	2 (1.8)	27 (29.3)	2 (1.6)	4 (10.8)	35 (9.5)
Healthy weight (18.5–24.9)	46 (40.4)	50 (54.3)	20 (16.0)	22 (59.5)	138 (37.5)
Overweight (25–29.9)	45 (39.5)	14 (15.2)	58 (46.4)	8 (21.6)	125 (34.0)
Obese (≥30)	18 (15.8)	1 (1.1)	44 (35.2)	3 (8.1)	66 (17.9)
Missing	3 (2.6)	0	1 (0.8)	0	4 (1.1)
Highest education completed, n (%)					
No formal education/Primary school incomplete	104 (91.2)	88 (95.7)	75 (60.0)	25 (67.6)	292 (79.3)
Primary school complete/Secondary school incomplete	4 (3.5)	4 (4.3)	46 (36.8)	7 (18.9)	61 (16.6)
Secondary school complete/Vocational/Some college/university	0	0	3 (2.4)	5 (13.5)	8 (2.2)
Missing	6 (5.3)	0	1 (0.8)	0	7 (1.9)
Occupation, n (%)					
Agriculture	0	69 (75.0)	22 (17.6)	29 (78.4)	120 (32.6)
Commercial	3 (2.6)	3 (3.3)	4 (3.2)	4 (10.8)	14 (3.8)
Household	110 (96.5)	11 (12.0)	92 (73.6)	1 (2.7)	214 (58.2)
Other	1 (0.9)	3 (3.3)	6 (4.8)	2 (5.4)	12 (3.3)
Unemployed/Missing	0	6 (6.5)	1 (0.8)	1 (2.7)	8 (2.2)
Minimum dietary diversity, n (%) ^c^					
High	3 (2.6)	0	30 (24.0)	0	33 (9.0)
Medium	25 (21.9)	4 (4.3)	73 (58.4)	8 (21.6)	110 (29.9)
Low	86 (75.4)	88 (95.7)	22 (17.6)	29 (78.4)	225 (61.1)
*Household and Exposure Characteristics*					
Household size					
Mean (SD)	7.6 (2.8)	4.4 (1.3)	5.6 (1.8)	6.1 (2.3)	6.0 (2.4)
Range	3–18	2–9	2–12	2–10	2–18
Primary fuel, n (%)					
Charcoal	0	0	0	1 (2.7)	1 (0.3)
Cow dung	0	0	110 (88.0)	0	110 (29.9)
Wood	114 (100)	92 (100)	11 (8.8)	35 (94.6)	252 (68.5)
Other/Missing	0	0	4 (3.2)	1 (2.7)	5 (1.4)
Some in the household smokes, n (%)					
No	102 (89.5)	61 (66.3)	125 (100)	33 (89.2)	321 (87.2)
Yes	12 (10.5)	31 (33.7)	0	3 (8.1)	46 (12.5)
Missing	0	0	0	1 (2.7)	1 (0.3)
Household assets owned, n (%)					
Color television	59 (51.8)	69 (75.0)	70 (56.0)	3 (8.1)	201 (54.6)
Radio	50 (43.9)	19 (20.7)	98 (78.4)	15 (40.5)	182 (49.5)
Mobile phone	111 (97.4)	75 (81.5)	124 (99.2)	27 (73.0)	337 (91.6)
Bicycle	15 (13.2)	13 (14.1)	61 (48.8)	11 (29.7)	100 (27.2)
Bank account	40 (35.1)	82 (89.1)	36 (28.8)	9 (24.3)	167 (45.4)
Access to electricity, n (%)					
No	7 (6.1)	3 (3.3)	8 (6.4)	23 (62.2)	41 (11.1)
Yes	107 (93.9)	89 (96.7)	117 (93.6)	11 (29.7)	324 (88.0)
Missing	0	0	0	3 (8.1)	3 (0.8)
Household food insecurity, n (%) ^d^					
Moderate/Severe	12 (10.5)	4 (4.3)	18 (14.4)	16 (43.2)	50 (13.6)
Mild	36 (31.6)	15 (16.3)	51 (40.8)	10 (27.0)	112 (30.4)
None	64 (56.1)	73 (79.3)	53 (42.4)	11 (29.7)	201 (54.6)
Missing	2 (1.8)	0	3 (2.4)	0	5 (1.4)

Note.

a Summary based on 368 adult women participants who had at least one BP measurement.

b The body mass index (BMI) is calculated as a person’s weight (in kilograms) divided by the square of height in meters.

c The minimum dietary diversity score is derived from the Minimum Dietary Diversity for Women (MDD-W) questionnaire, which we adapted to cover a 30-day reference period. In the MDD-W, minimum dietary diversity is defined as consuming at least 5 of 10 food groups in the previous day(HAPIN Investigators).

d Household food insecurity is measured by the Food Insecurity Experience Scale (FIES), which was applied with a 30-day reference period. In the FIES, higher scores represent increasingly severe food insecurity (Glymour et al., 2005).

**Table 2:** Results of change-score^a^ analyses for PM_2.5_, BC, and CO^b^ and blood pressure measures.

		PM_2.5_ (μg/m^3^, n = 213)			BC (μg/m^3^, n = 202)			CO (ppm, n = 201)		
		Estimate	SE	*p*	Estimate	SE	*p*	Estimate	SE	*p*
SBP (mmHg)	Linear	−0.009	0.011	0.44	−0.047	0.143	0.74	−0.626	0.436	0.15
	Log Linear	−0.828	1.078	0.44	−0.871	1.187	0.46	1.050	0.736	0.16
DBP (mmHg)	Linear	0.009	0.010	0.36	0.177	0.118	0.13	−0.458	0.353	0.20
	Log Linear	0.951	0.898	0.29	1.217	0.981	0.22	0.151	0.599	0.80

Note.

a Assuming a higher blood pressure due to higher pollutant exposures might lead to increased BP over time, this difference (baseline-final BP) would be expected to be negative. The analysis was restricted to those with a final last visit. Models are adjusted for age, education, diet diversity, BMI, food insecurity, IRC, and time of day.

b Time-weighted average exposures. For the control group, an average was calculated from all available measurements. For the intervention group, the baseline level is weighted by time to randomization, and the average thereafter is weighted by time after randomization.

**Table 3:** Mixed models^a^ with average effect of time-weighted mean pollutant exposure on BP (long-term effect) and average short-term effect of the visit-specific pollutant on BP.

		PM_2.5_ (μg/m^3^, n = 1395)			BC (μg/m^3^, n = 1123)			CO (ppm, n = 1198)		
		Estimate	ICC	*p*	Estimate	ICC	*p*	Estimate	ICC	*p*
SBP (mmHg)	Long-term^b^	0.00165	0.60	0.85	0.108	0.59	0.27	0.356	0.60	0.29
	Short-term^c^	0.00353		0.15	0.0864		**0.01**	0.0267		0.80
DBP (mmHg)	Long-term^b^	−0.00240	0.51	0.66	−0.0133	0.52	0.84	0.117	0.52	0.59
	Short-term^c^	0.00202		0.27	0.0545		**0.03**	0.0368		0.64

Note.

a Adjusted for age, education, diet diversity, BMI, food insecurity, time of day, and IRC.

b Time-weighted mean pollutant level over follow-up (long-term effect).

c Average change from mean (short-term effect).

**Table 4:** Mixed models^a^ for short-term pollutant effect for PM_2.5_ and BC.

		PM_2.5_ (n = 1444)				BC (n = 1298)			
		Estimate	ICC	AIC	*p*	Estimate	ICC	AIC	*p*
SBP (mmHg)	Linear	0.00353	0.59	10951	0.13	0.0914	0.59	9961	**0.005**
	Log Linear	0.530		10941	0.07	0.953		9856	**0.004**
	Quartile 2^b^	−0.221		10934	0.78	−0.490		9849	0.56
	Quartile 3^b^	0.744			0.48	1.46			0.11
	Quartile 4^b^	1.601			**0.05**	1.96			**0.03**
DBP (mmHg)	Linear	0.00155	0.51	9981	0.37	2	0.52	8980	**0.05**
	Log Linear	0.109		9972	0.61	2.11		8975	**0.04**
	Quartile 2^b^	0.221		9968	0.70	0.48		8972	0.63
	Quartile 3^b^	0.0836			0.89	1.49			0.14
	Quartile 4^b^	0.417			0.49	1.72			0.09

Note.

ICC = intraclass correlation coefficient.

AIC = Akaike information criterion.

a Adjusted for age, education, diet diversity, BMI, food insecurity, time of day, and IRC.

b Quartile 1 is the referent.

## Data availability

Data will be made available on request.

## Appendix A. Supplementary data

Supplementary data to this article can be found online at https://doi.org/10.1016/j.envres.2025.122570.
