# Supplementary material for: Household air pollution and blood pressure among adult women participants of the Household Air Pollution Intervention Network Trial: An exposure-response analysis
Source: Environ Res. Author manuscript; Available in PMC 2026 May 12. (PMC13166104; doi:10.1016/j.envres.2025.122570)
Supplement: Pillarisetti_EnvRes_2025_SI [file NIHMS2165636-supplement-Pillarisetti_EnvRes_2025_SI.docx]

**Supplementary Information**

Supplement for Ye W, Steenland K, Pillarisetti A, et al. Household air pollution and blood pressure among adult women participants of the Household Air Pollution Intervention Network Trial: An exposure-response analysis.

[Table S1. A priori covariate adjustments in exposure-response analyses 3](#_Toc203362287)

[Table S2. Descriptive statistics by study period. 3](#_Toc203362288)

[Table S3. Number (%) in each blood pressure category 4](#_Toc203362289)

[Table S4. Results of change-score quartile^#^ analyses^*^ for PM_2.5_, BC, and CO^**^ and blood pressure 4](#_Toc203362290)

[Table S5. Analyses stratified by Age (at median), BMI (at median), and IRC for PM2.5 and BC and SBP 5](#_Toc203362291)

[Table S6. Linear model for Mean Arterial Pressure and Pulse Pressue: change score model 6](#_Toc203362292)

[Table S7. Linear model for Mean Arterial Pressure and Pulse Pressure: change score model 6](#_Toc203362293)

[Table S8. Comparison table for personal exposure, BP measurements and exposure-response relationship with select studies 7](#_Toc203362294)

[Table S9. Comparison table for personal exposure, BP measurements and exposure-response relationship with select studies 8](#_Toc203362295)

[Figure S1. Line plot of SBP and DBP by study arm and visit. 10](#_Toc203362296)

[Figure S2. Line plot of SBP and DBP by study arm and visit in Guatemala. 10](#_Toc203362297)

[Figure S3. Line plot of SBP and DBP by study arm and visit in India. 11](#_Toc203362298)

[Figure S4. Line plot of SBP and DBP by study arm and visit in Peru. 11](#_Toc203362299)

[Figure S5. Line plot of SBP and DBP by study arm and visit in Rwanda. 12](#_Toc203362300)

[Figure S6. Baseline and intervention stoves. Photos of stoves from (A) Guatemala, (B) India, (C) Peru, and (D) Rwanda. 13](#_Toc203362301)

[References 14](#_Toc203362302)

**Table S1. A priori covariate adjustments in exposure-response analyses**

| Parameter | Type | Subgroup Definitions |
| --- | --- | --- |
| International Research Center | Categorical | Guatemala, India, Peru, Rwanda |
| Baseline: Age at baseline (years) | Continuous | Calculated as the date at baseline minus the date of birth. Date at baseline is assigned by the date of visit if not missing. |
| Baseline: Highest level of education completed | Categorical | No formal education or some primary school  Primary school or some secondary school incomplete  Secondary school or vocational or university/college  Missing |
| Baseline: Body mass index (BMI) | Continuous | BMI calculated as the average weight (kg) divided by the average height squared (m^2^) |
| Baseline: Household food insecurity score | Categorical | Categories (corresponding score):  Food secure (0)  Mild (1,2,3)  Moderate (4,5,6) / Severe (7,8)  Missing (34 missing across all visits  See [**http://www.fao.org/3/as583e/as583e.pdf**](http://www.fao.org/3/as583e/as583e.pdf) |
| Baseline: Minimum diet diversity | Categorical | Categories (corresponding diet diversity score):  Low (< 4)  Medium (4-5)  High (>5)  Missing (33 missing across all vists)  See [**https://inddex.nutrition.tufts.edu/data4diets/indicator/minimum-dietary-diversity-women-mdd-w**](https://inddex.nutrition.tufts.edu/data4diets/indicator/minimum-dietary-diversity-women-mdd-w) |
| Time of the blood pressure measurement; time-varying | Categorical | AM or PM |

# Table S2. Descriptive statistics by study period.

|  | **SBP** | **DBP** | **PP** | **MAP** |
| --- | --- | --- | --- | --- |
| ***Baseline*** |  |  |  |  |
| Average (SD) | 114.6 (15.1) | 69.7 (10.6) | 45 (8.8) | 84.7 (11.5) |
| Median [IQR] | 112.8 (18.7) | 69 (14.5) | 43.7 (10) | 83.5 (15) |
| Range | 81-187.3 | 38.3-106.3 | 26.7-83.3 | 54.9-133.3 |
| ***Post-Intervention*** |  |  |  |  |
| Average (SD) | 111.7 (12.8) | 68.4 (9.1) | 43.3 (7.4) | 82.8 (9.9) |
| Median [IQR] | 109.5 (15.3) | 67.3 (13.3) | 41.9 (7.5) | 81.2 (13.6) |
| Range | 87-177.9 | 48.9-101 | 28.8-83.3 | 62.6-122.3 |

# *SBP: Systolic Blood Pressure; DBP: Diastolic Blood Pressure; PP: Pulse Pressure; MAP: Mean Arterial Pressure*

# Table S3. Number (%) in each blood pressure category

| **BP Category*, n (%)** | **Baseline** | | **Pregnancy** | | **Post-birth** | |
| --- | --- | --- | --- | --- | --- | --- |
|  | Control | Intervention | Control | Intervention | Control | Intervention |
| Normal blood pressure | 121 (66.1) | 116 (64.8) | 210 (72.4) | 220 (74.3) | 248 (72.9) | 247 (73.1) |
| Elevated blood pressure | 20 (10.9) | 28 (15.6) | 27 (9.3) | 24 (8.1) | 31 (9.1) | 25 (7.4) |
| Hypertension Stage 1 | 29 (15.8) | 20 (11.2) | 40 (13.8) | 32 (10.8) | 43 (12.6) | 47 (13.9) |
| Hypertension Stage 2 | 13 (7.1) | 15 (8.4) | 13 (4.5) | 20 (6.8) | 18 (5.3) | 19 (5.6) |
| **Total** | 183 (100) | 179 (100) | 290 (100) | 296 (100) | 340 (100) | 338 (100) |

*Note:*

**American Heart Association blood pressure category: normal blood pressure (SBP < 120 mmHg and DBP < 80 mmHg); elevated blood pressure (SBP between 120-129 mmHg and DBP < 80 mmHg); hypertension stage 1 (SBP between 130-139 mmHg or DBP between 80-89 mmHg; hypertension stage 2 (SBP >= 140 mmHg or DBP >= 90 mmHg).*

# Table S4. Results of change-score quartile^#^ analyses^*^ for PM_2.5_, BC, and CO^**^ and blood pressure

|  |  | **PM_2.5_** | | **BC** | | **CO** | |
| --- | --- | --- | --- | --- | --- | --- | --- |
|  |  | Estimate | SE | Estimate | SE | Estimate | SE |
| SBP | Q2 | -1.832 | 2.458 | 2.002 | 2.506 | -0.649 | 2.396 |
|  | Q3 | -2.271 | 2.465 | -2.013 | 2.480 | 2.776 | 2.451 |
|  | Q4 | -1.373 | 2.433 | -0.908 | 2.559 | -3.823 | 2.583 |
| DBP | Q2 | 1.121 | 2.046 | 2.587 | 2.073 | 1.285 | 1.947 |
|  | Q3 | -0.302 | 2.051 | -0.063 | 2.052 | 1.664 | 1.992 |
|  | Q4 | 2.373 | 2.025 | 2.740 | 2.117 | 0.214 | 2.099 |

* higher BP due to higher pollutant might lead to increased BP over time; difference would be negative. Restricted to those with last visit. Adjusted for age, education, diet diversity, BMI, food insecurity, time of day

** average pollutant level over follow-up; for intervention group baseline level weighted by time to randomization, average thereafter weighted by time after randomization

^#^ all quartiles vs. lowest category as reference

# Table S5. Analyses stratified by Age (at median), BMI (at median), and IRC for PM2.5 and BC and SBP

A. Change-score models

|  |  |  | **PM_2.5_** | | **BC** | | |
| --- | --- | --- | --- | --- | --- | --- | --- |
|  |  | Estimate | SE | *p* | Estimate | SE | *p* |
| Age | ≤51 | 0.003 | 0.017 | 0.85 | 0.134 | 0.17 | 0.44 |
|  | >51 | -0.06 | 0.017 | 0.14 | -0.34 | 0.27 | 0.2 |
| BMI | ≤25 | -0.024 | 0.033 | ..28 | -0.24 | 0.25 | 0.33 |
|  | >25 | -0.006 | 0.013 | 0.66 | 0.012 | 0.17 | 0.95 |
| IRC* | Guatemala | -0.059 | 0.19 | 0.002 | -0.91 | 0.27 | 0.002 |
|  | India | -0.015 | 0.017 | 0.38 | -0.12 | 0.23 | 0.93 |
|  | Peru | 0.045 | 0.29 | 0.12 | 0.3 | 0.29 | 0.3 |

*too few observations for Rwanda to estimate change score effect

B. Mixed effects models (long-term)

|  |  |  | **PM_2.5_** | | **BC** | | |
| --- | --- | --- | --- | --- | --- | --- | --- |
|  |  | Estimate | SE | *p* | Estimate | SE | *p* |
| Age | ≤51 | 0.0053 | 0.0108 | 0.63 | 0.0909 | 0.0991 | 0.36 |
|  | >51 | -0.0047 | 0.0141 | 0.74 | 0.1447 | 0.225 | 0.64` |
| BMI | ≤25 | -0.0188 | 0.014 | 0.19 | 0.0751 | 0.1855 | 0.68 |
|  | >25 | 0.0173 | 0.0107 | 0.11 | 0.1707 | 0.115 | 0.15 |
| IRC | Rwanda |  |  |  |  |  |  |
|  | Guatemala | -0.0152 | 0.0206 | 0.46 | 0.0818 | 0.3439 | 0.81 |
|  | Peru | 0.027 | 0.0085 | 0.002 | 0.2 | 0.0982 | 0.04 |
|  | India | -0.0388 | 0.0201 | 0.054 | -0.0314 | 0.1996 | 0.87 |

C. Mixed effects models (short-term)

|  |  |  | **PM_2.5_** | | **BC** | | |
| --- | --- | --- | --- | --- | --- | --- | --- |
|  |  | Estimate | SE | *p* | Estimate | SE | *p* |
| Age | ≤51 | 0.0020 | 0.0029 | 0.63 | 0.0600 | 0.040 | 0.13 |
|  | >51 | 0.0057 | 0.0043 | 0.19 | 0.126 | 0.0589 | 0.03 |
| BMI | ≤25 | 0.0046 | 0.0038 | 0.19 | 0.1399 | 0.0589 | 0.01 |
|  | >25 | 0.0028 | 0.0031 | 0.37 | 0.0502 | 0.0391 | 0.20 |
| IRC | Rwanda | 0.0078 | 0.0118 | 0.51 | 0.087 | 0.1739 | 0.62 |
|  | Guatemala | 0.0022 | 0.0046 | 0.63 | 0.0707 | 0.0768 | 0.36 |
|  | Peru | 0.00154 | 0.0035 | 0.66 | 0.0518 | 0.0447 | 0.25 |
|  | India | 0.0064 | 0.0053 | 0.23 | 0.1263 | 0.0665 | 0.06 |

# Table S6. Linear model for Mean Arterial Pressure (MAP) and Pulse Pressure (PP): change score model

| **PM2.5** | **estimate** | **std err** | **t value** | **p value** | **n** | **R sq** |
| --- | --- | --- | --- | --- | --- | --- |
| MAP | 0.003 | 0.009 | 0.320 | 0.7493 | 213 | 0.11 |
| PP | 0.018 | 0.009 | -1.890 | 0.0597 | 213 | 0.08 |
| **BC** |  |  |  |  |  |  |
| MAP | 0.102 | 0.114 | 0.900 | 0.3709 | 202 | 0.11 |
| PP | -0.223 | 0.118 | -1.900 | 0.0589 | 202 | 0.08 |
| **CO** |  |  |  |  |  |  |
| MAP | -0.514 | 0.347 | -1.48 | 0.1394 | 201 | 0.12 |
| PP | -0.168 | 0.344 | -0.490 | 0.6264 | 201 | 0.07 |

# Table S7. Linear model for Mean Arterial Pressure (MAP) and Pulse Pressure (PP): change score model

| **Repeated measures with both long term and short term exposure in model** | | | | | |
| --- | --- | --- | --- | --- | --- |
| **PM2.5** | **estimate** | **std err** | **t value** | **p value** | **n** |
| MAP average | -0.00097 | 0.006195 | -0.16 | 0.8753 | 1395 |
| MAP change from avg | 0.002519 | 0.00187 | 1.35 | 0.1 |  |
| PP average | 0.00372 | 0.005162 | 0.72 | 0.4713 | 1395 |
| PP change from avg | 0.001441 | 0.001811 | 0.80 | 0.4265 |  |
| **BC** | | | | | |
| MAP average | 0.02819 | 0.07204 | 0.39 | 0.6956 | 1197 |
| MAP change from avg | 0.06551 | 0.02555 | 2.56 | 0.0105 |  |
| PP average | 0.1178 | 0.06066 | 1.94 | 0.0524 | 1197 |
| PP change from avg | 0.03104 | 0.02523 | 1.23 | 0.2189 |  |
| **CO** | | | | | |
| MAP average | 0.1954 | 0.2442 | 0.8 | 0.4239 | 1236 |
| MAP change from avg | 0.03374 | 0.08077 | 0.42 | 0.6762 |  |
| PP average | 0.238 | 0.2032 | 1.17 | 0.2418 | 1236 |
| PP change from avg | -0.00971 | 0.07753 | -0.13 | 0.9 |  |
|  | | | | | |
| **Repeated measures with short term exposure in model** | | | | | |
| **PM2.5** | | | | | |
| MAP | 0.002252 | 0.001776 | 1.27 | 0.2052 | 1444 |
| PP | 0.001925 | 0.001706 | 1.13 | 0.2594 |  |
| **BC** | | | | | |
| MAP | 0.06258 | 0.02 | 2.6 | 0.0094 | 1298 |
| PP | 0.04689 | 0.02 | 2.01 | 0.045 |  |
| **CO** | | | | | |
| MAP | 0.04123 | 0.08 | 0.55 | 0.5839 | 1325 |
| PP | 0.04623 | 0.07204 | 0.64 | 0.5211 |  |

# Table S8. Comparison table for personal exposure, BP measurements and exposure-response relationship with select studies

*We do not have data on temperature to enable us to control for this on a daily basis. We have added this to the limitations noted in the Discussion (~L328). In our data, we are able to distinguish winter months from other months. Cold months predicted higher BP in our adjusted model, but t-tests found no significant differences in either PM2.5 or BC levels during winter months. As a supplemental analysis, we re-ran models including a variable for winter (defined separately for each country). Including the winter term had little effect on the associations with exposure (see below; for example, an ~2% decrease in the coefficient for BC). We have noted this in the text (~L313—317) and included the table in the SI.*

|  |  | *PM_2.5_ (µg/m^3^)* | | *BC (µg/m^3^)* | | *CO (ppm)* | |
| --- | --- | --- | --- | --- | --- | --- | --- |
|  |  | *Estimate* | *p* | *Estimate* | *p* | *Estimate* | *p* |
| *SBP (mmHg)* | *Long-term* | *0.001385* | *0.87* | *0.1080* | *0.27* | *0.3459* | *0.30* |
|  | *Short-term* | *0.003270* | *0.19* | *0.08439* | *0.01* | *0.01705* | *0.87* |
| *DBP (mmHg)* | *Long-term* | *-0.00192* | *0.77* | *-0.01334* | *0.84* | *0.1081* | *0.61* |
|  | *Short-term* | *0.001646* | *0.36* | *0.05368* | *0.03* | *0.02941* | *0.70* |

# Table S9. Comparison table for personal exposure, BP measurements and exposure-response relationship with select studies

| **Study and Participants** | **24-hr Exposure Level** | **BP Level** | **Exposure-Response Relationship** |
| --- | --- | --- | --- |
| CLEAN-Air (Africa)^1^ – cross-sectional  Ghana (n = 70)  Adult women/men, mean age 35 yrs. | PM_2.5_, GM (95% CI)  Overall: 54.4 (44.3, 67.4)  Solid fuel users: 65.6 (52.2, 83.5)  LPG users: 45.9 (33.3, 65.1) | SBP (Mean, SD) mmHg  Overall: 117.9 (15.8)  Solid fuel users: 119.6 (17.1)  LPG users: 116.1 (14.2)  DBP (Mean, SD) mmHg  Overall: 78.0 (11.7)  Solid fuel users: 78.5 (12.4)  LPG users: 77.5 (11.0) | A 1-log-µg/m^3^ increase in PM_2.5_ exposure was associated with -2.42 (95% CI: -8.65, 3.80) mmHg SBP and -0.28 (95% CI: -5.04, 4.48) mmHg DBP. |
| INTERMAP China Prospective^2^ – longitudinal  China (n = 753)  Adult women/men, mean age 62.8 yrs. | PM_2.5_, (Mean, SD) µg/m^3^  Yearly exposure: 97.5 (79.2) | SBP (Mean, SD) mmHg  138.9 (165) – 125.9 (16.2)  DBP (Mean, SD) mmHg  70.7 (9.7) – 79.1 (10.7) | A 1-ln-µg/m^3^ increase in PM_2.5_ exposure was associated with 1.5 (95% CI: 0.2, 2.7) mmHg SBP and 1.0 (95% CI: 0.4, 1.7) mmHg DBP. |
| CHAP^3^ – longitudinal  Peru (n = 180)  Adult women, mean age 48 yrs. | PM_2.5_, Mean, (SD) µg/m^3^  Control: 126 (214)  Intervention: 104 (100)  BC, Mean (SD) µg/m^3^  Control: 19 (17)  Intervention: 21 (22)  CO, Mean (SD) ppm  Control: 6.6 (8.2)  Intervention: 7.1 (8.4) | SBP (Mean, SD) mmHg  Control: 102.3 (11)  Intervention: 100.4 (11.9)  DBP (Mean, SD) mmHg  Control: 68.1 (8.3)  Intervention: 67.7 (8.7) | No consistent exposure-response relationships between PM/BC/CO and BP. |
| Young et al. (2018)^4^ – cross-sectional  Honduras (n = 147)  Adult women, mean age 37.0 yrs. | PM_2.5_, Mean (SD) µg/m^3^  Overall: 101 (70)  Traditional stoves: 126 (77)  *Justa* stoves: 66 (38)  BC, Mean (SD) µg/m^3^  Overall: 17 (22)  Traditional stoves: 24 (26)  *Justa* stoves: 7 (8) | SBP (Mean, SD) mmHg  Overall: 118.3 (12.4)  Traditional stoves: 119.9 (11.6)  *Justa* stoves: 116.6 (13.0)  DBP (Mean, SD) mmHg  Overall: 73.1 (8.7)  Traditional stoves: 73.8 (8.5)  *Justa* stoves: 72.3 (8.9) | A 1-log-µg/m^3^ increase in personal PM_2.5_ exposure was associated with 0.8 (95% CI: -2.2, 3.8) mmHg SBP and 0.4 (95% CI: -2.0 to 2.7) mmHg DBP.  A 1-log-µg/m^3^ increase in personal BC exposure was associated with 0.5 (95% CI: -1.0, 2.0) mmHg SBP and 0.03 (95% CI: -1.2 to 1.2) mmHg DBP. |
| Baumgartner et al. (2018)^5^ – longitudinal  China, Sichuan (n = 205)  Adult women, mean age 51.9 yrs. | PM_2.5_, Mean (SD), Median µg/m^3^  Winter: 218.5 (202.3), 151.7  Summer: 101.3 (98.3), 75.5  BC, Mean (SD), Median µg/m^3^  Winter: 9.2 (15.2), 4.1  Summer: 4.4 (8.6), 2.6 | SBP Mean (SD), Median [mmHg]  Winter: 127.2 (18.5), 123.5  Summer: 120.7 (22.2), 114.0  DBP Mean (SD), Median [mmHg]  Winter: 77.9 (10.0), 77.5  Summer: 73.5 (10.6), 72.0 | A 1-ln-µg/m^3^ increase in PM_2.5_ exposure was associated with higher SBP (brachial: 2.4 mm Hg; central: 2.4 mm Hg) and PP (peripheral: 1.8 mm Hg; central: 1.8 mm Hg), but not DBP.  BC exposure had a similar magnitude of association with SBP and PP as PM_2.5_ and was also associated with higher DBP.  Associations are generally larger in older (>50 yrs.) and in summer. |
| Baumgartner et al. (2014 and 2011) – longitudinal  China, Yunnan (n = 280)  Adult women, mean age 51.9 yrs. | PM_2.5_, GM (95% CI)  Winter: 117 (107, 128)  Summer: 55 (49-62)  BC, GM (range)  Winter: 6 (6, 7)  Summer: 4 (4, 4) | SBP (Mean, 95% CI) mmHg  120 (118, 122)  DBP (Mean, 95% CI) mmHg  72 (71, 73) | A 1-ln-μg/m^3^ increase in BC was associated with 4.3 (2.3, 63) mmHg higher SBP and 1.3 (0.2, 2.4) mmHg DBP.  A 1-ln-μg/m^3^ increase in PM_2.5_ was associated with 2.2 (0.8, 3.6) mmHg higher SBP and 0.5 (-0.3, 1.3) mmHg DBP.  A 1-ln-μg/m^3^increase in BC exposure was associated with a 1.8 mmHg (95% CI, 0 to 3.6) higher SBP in younger women at the sample average, compared with no effect for PM_2.5_.  Among women>50 y old, a 1-ln-μg/m^3^ increase in BC was associated with a 7.4 mmHg (95% CI, 4.0 to 10.8) higher SBP and a 2.9 mmHg (95% CI, 1.1 to 4.7) higher DBP.  Among women > 50 years of age, a 1‐log‐μg/m3 increase in PM2.5 exposure was associated with 4.1 mm Hg higher SBP (95% CI, 1.5 to 6.6; p = 0.002) and 1.8 mm Hg higher DBP (95% CI, 0.4 to 3.2; p = 0.01). |


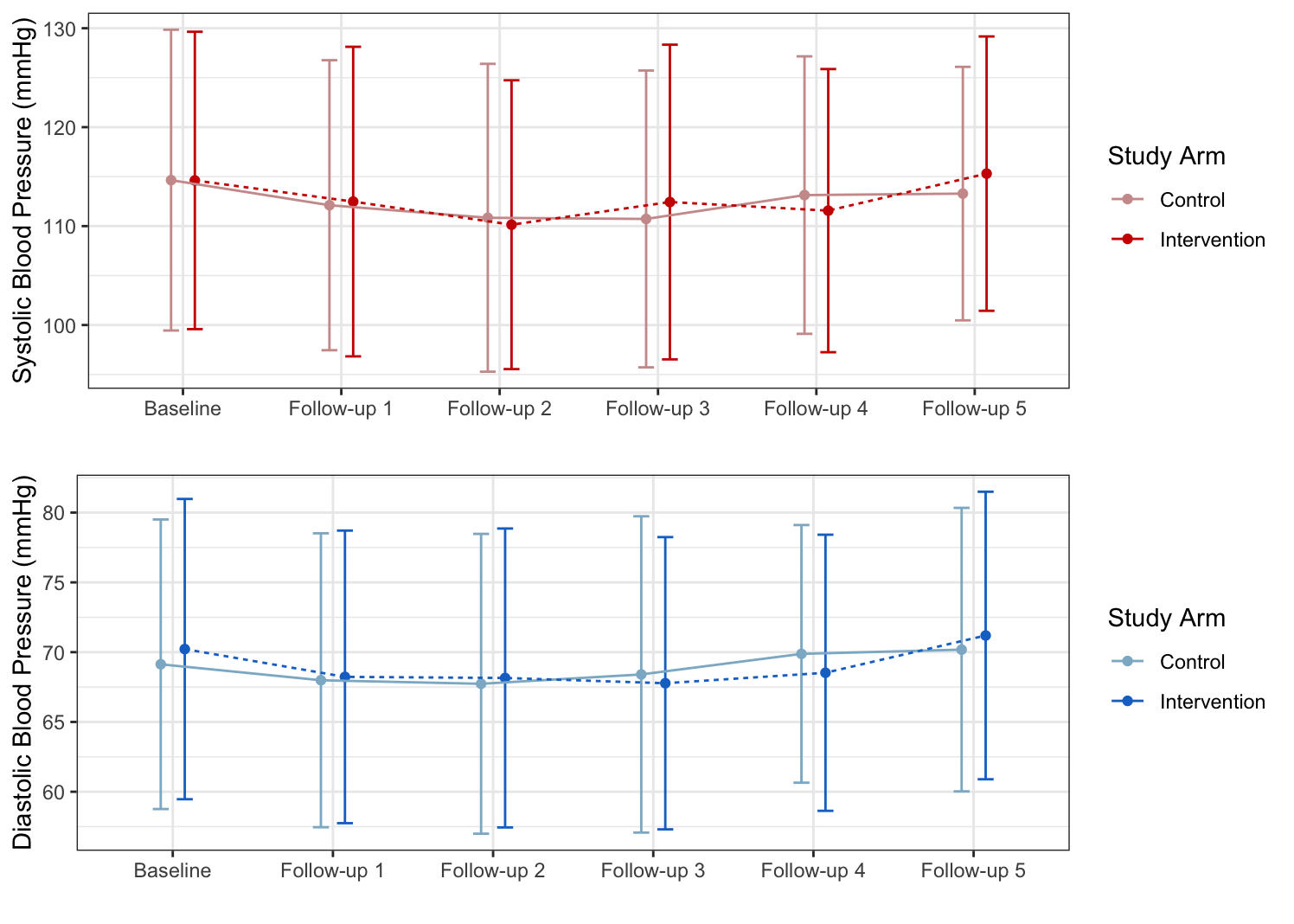


Figure S1. Line plot of SBP and DBP by study arm and visit. Dots indicate mean and error bars indicate one SD.

# Figure S2. Line plot of SBP and DBP by study arm and visit in Guatemala.

Dots indicate mean and error bars indicate one SD.

# Figure S3. Line plot of SBP and DBP by study arm and visit in India.

Dots indicate mean and error bars indicate one SD.

Figure S4. Line plot of SBP and DBP by study arm and visit in Peru.
Dots indicate mean and error bars indicate one SD.

Figure S5. Line plot of SBP and DBP by study arm and visit in Rwanda.
Dots indicate mean and error bars indicate one SD.

(A)


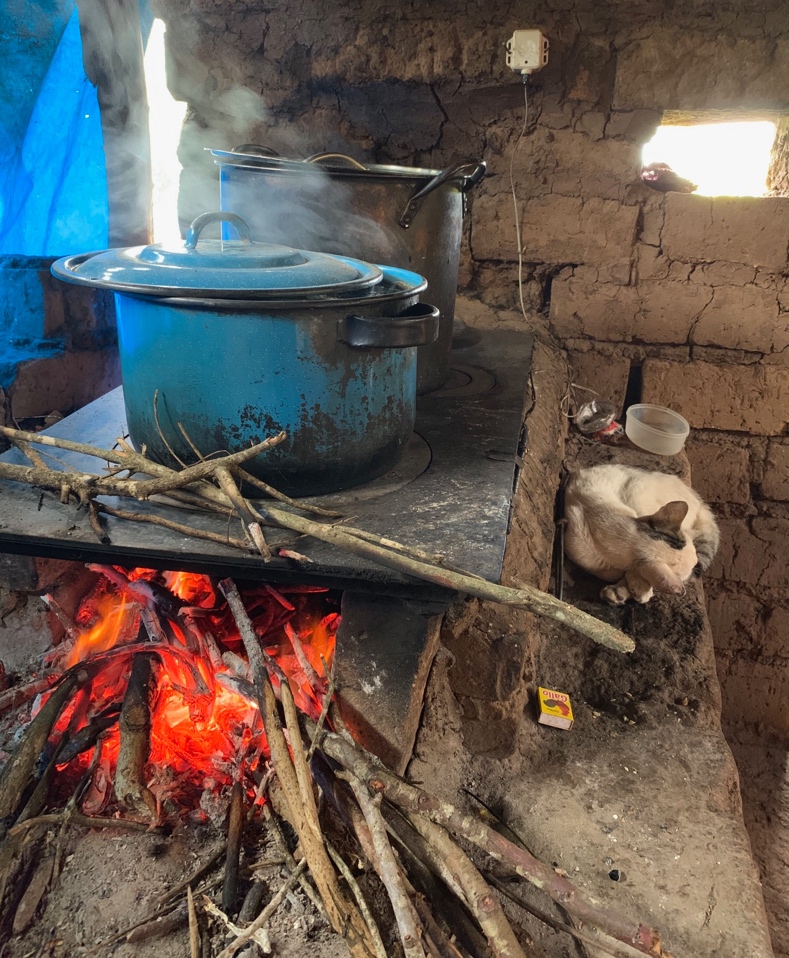

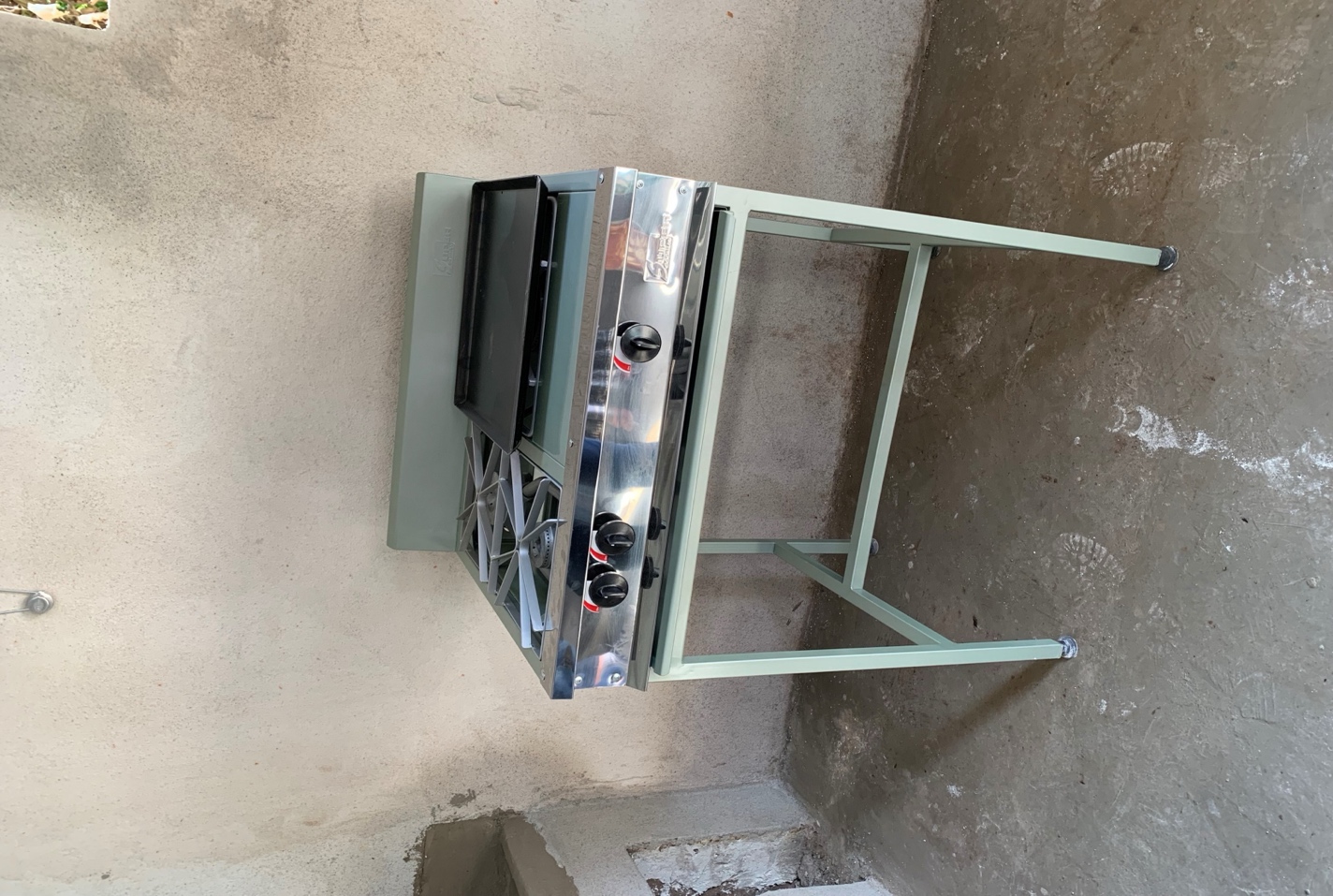


(B)


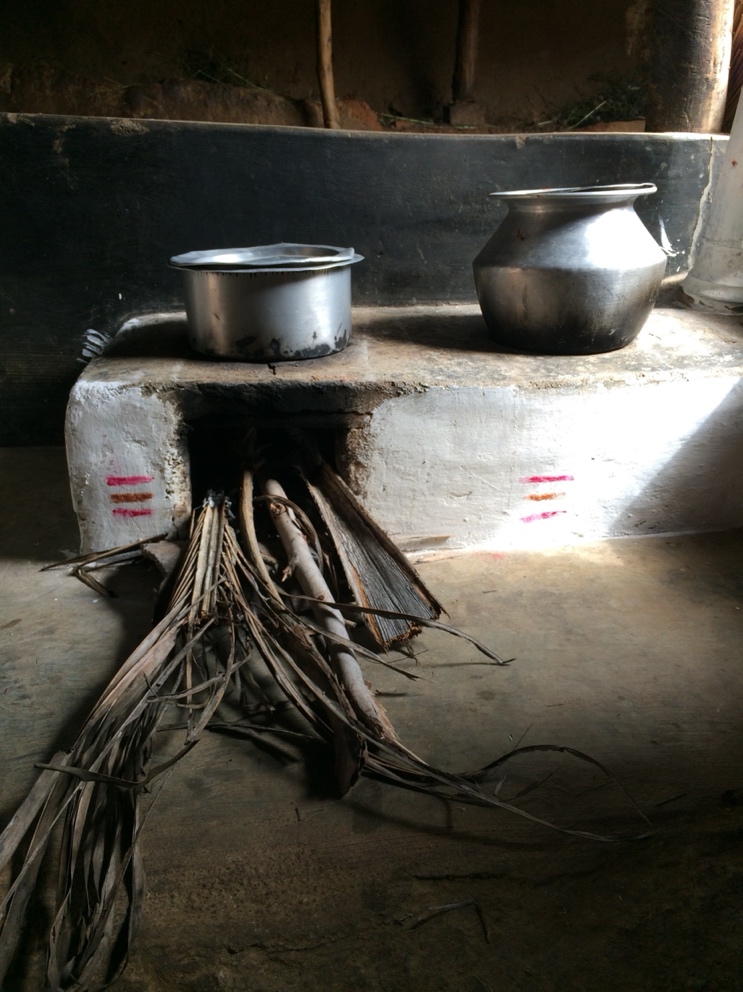

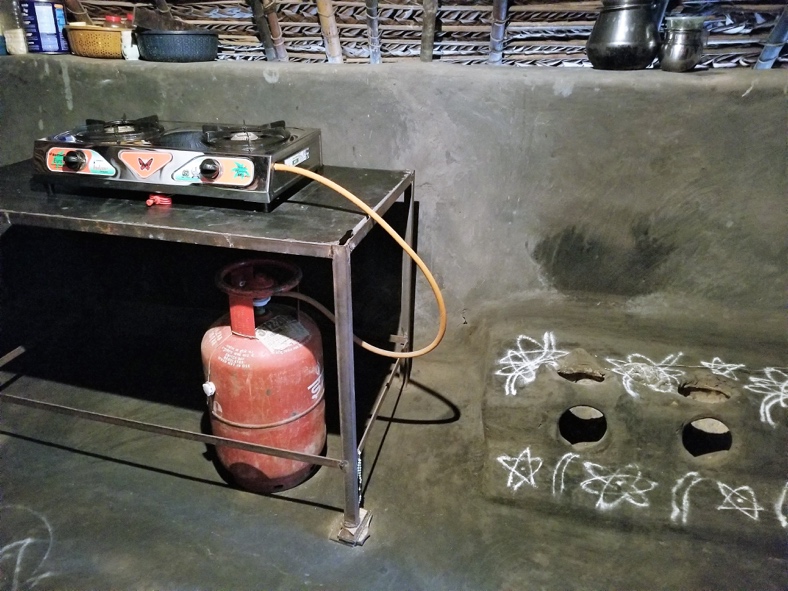


(C)


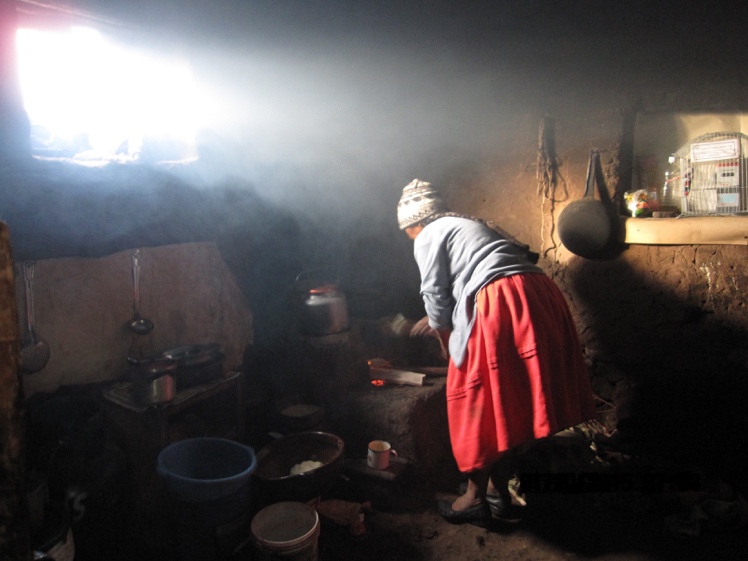

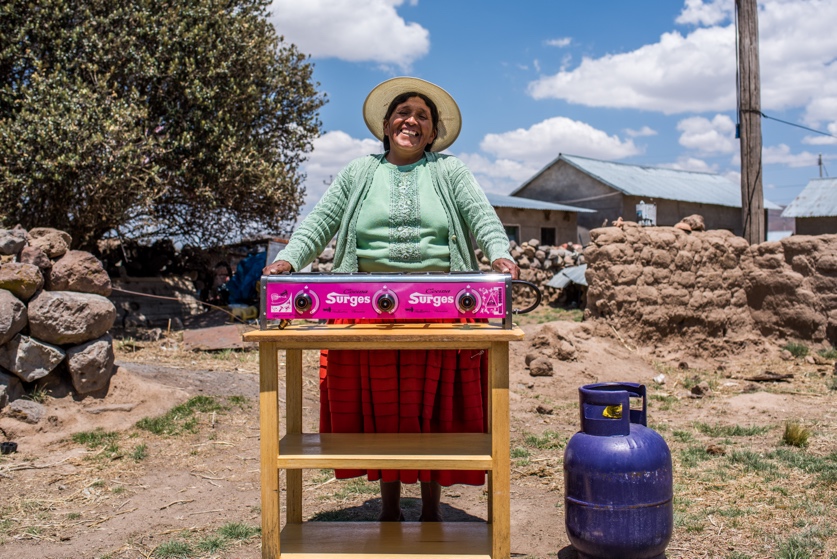


(D)


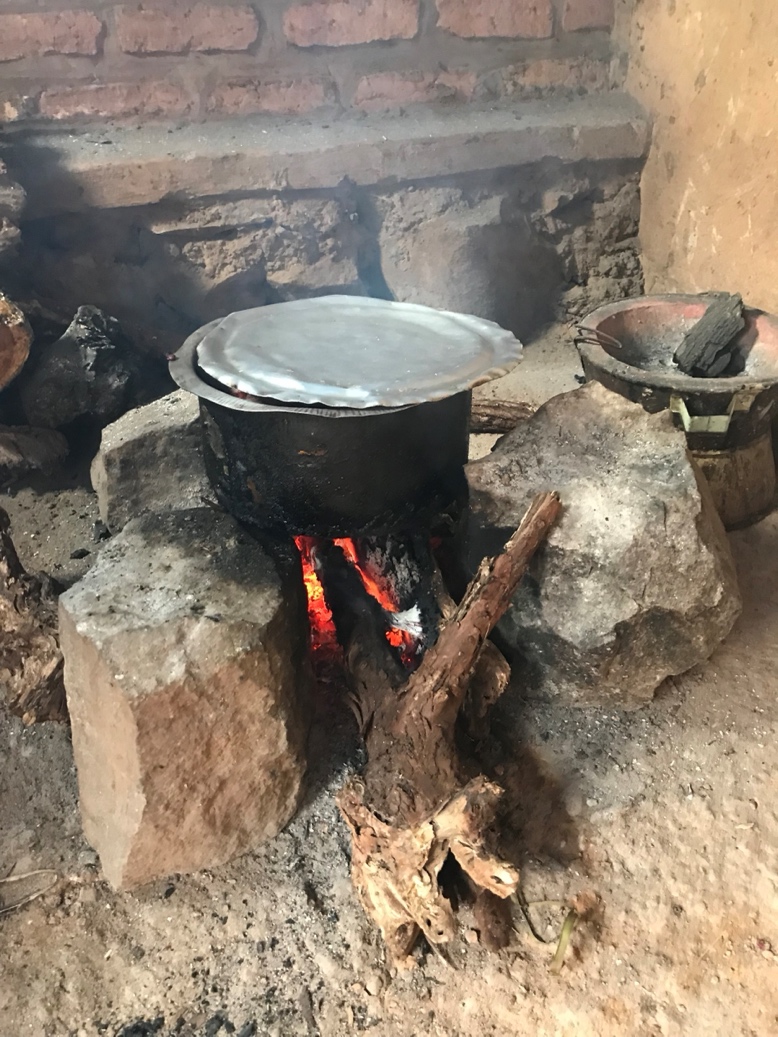

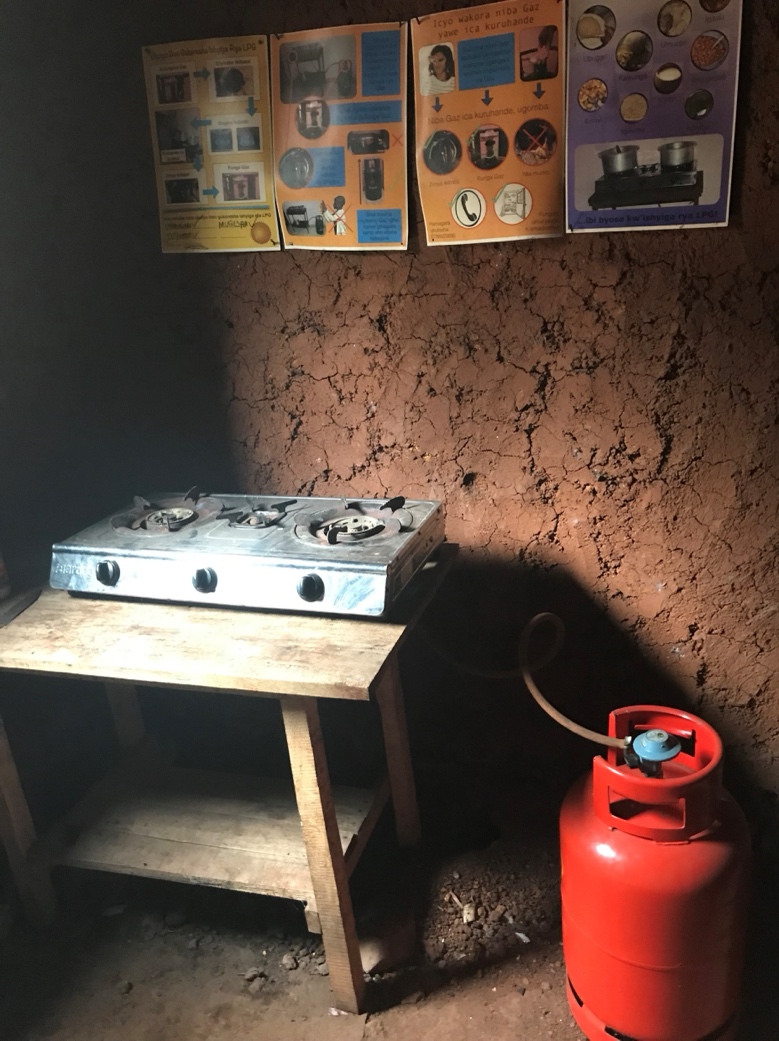


Figure S6. Baseline and intervention stoves. Photos of stoves from (A) Guatemala, (B) India, (C) Peru, and (D) Rwanda.

# References

1 Tawiah T, Shupler M, Gyaase S, *et al.* The Association between Household Air Pollution and Blood Pressure in Obuasi Municipality, Ghana. *Atmosphere* 2022; **13**: 2033.

2 Kanagasabai T, Xie W, Yan L, *et al.* Household Air Pollution and Blood Pressure, Vascular Damage, and Subclinical Indicators of Cardiovascular Disease in Older Chinese Adults. *American Journal of Hypertension* 2022; **35**: 121–31.

3 Checkley W, Williams KN, Kephart JL, *et al.* Effects of a Household Air Pollution Intervention with Liquefied Petroleum Gas on Cardiopulmonary Outcomes in Peru. A Randomized Controlled Trial. *Am J Respir Crit Care Med* 2021; **203**: 1386–97.

4 Young BN, Clark ML, Rajkumar S, *et al.* Exposure to household air pollution from biomass cookstoves and blood pressure among women in rural Honduras: A cross-sectional study. *Indoor Air* 2019; **29**: 130–42.

5 Baumgartner J, Carter E, Schauer JJ, *et al.* Household air pollution and measures of blood pressure, arterial stiffness and central haemodynamics. *Heart* 2018; **104**: 1515–21.
